# Malaria Elimination: Time to Target All Species

**DOI:** 10.4269/ajtmh.17-0869

**Published:** 2018-05-14

**Authors:** Andrew A. Lover, J. Kevin Baird, Roly Gosling, Ric N. Price

**Affiliations:** 1 Malaria Elimination Initiative at the University of California, San Francisco, San Francisco, California;; 2 Eijkman-Oxford Clinical Research Unit, Eijkman Institute of Molecular Biology, Jakarta, Indonesia;; 3 Centre for Tropical Medicine and Global Health, Nuffield Department of Medicine, University of Oxford, Oxford, United Kingdom;; 4 Global and Tropical Health Division, Menzies School of Health Research and Charles Darwin University, Darwin, Australia

## Abstract

Important strides have been made within the past decade toward malaria elimination in many regions, and with this progress, the feasibility of eradication is once again under discussion. If the ambitious goal of eradication is to be achieved by 2040, all species of *Plasmodium* infecting humans will need to be targeted with evidence-based and concerted interventions. In this perspective, the potential barriers to achieving global malaria elimination are discussed with respect to the related diversities in host, parasite, and vector populations. We argue that control strategies need to be reorientated from a sequential attack on each species, dominated by *Plasmodium falciparum* to one that targets all species in parallel. A set of research themes is proposed to mitigate the potential setbacks on the pathway to a malaria-free world.

## INTRODUCTION

In 2015, the United Nations with the Bill & Melinda Gates Foundation published a new framework for malaria eradication^1^; and the World Health Organization published a technical strategy for *Plasmodium vivax* elimination.^2^ Central to these documents is a combination of new strategies, tools, and financing mechanisms that will need to be delivered from a robust organizational structure that can act as a launchpad to ensure rapid acceleration of interventions and result in impact on the ground. If malaria elimination is to be achieved by 2040, the essential infrastructure of this launchpad will need to be created within the next 3–5 years, to allow sufficient time for implementation and scale-up toward impact. Because the significant progress observed in the last decade appears to have stalled in many endemic regions,^3^ the time is opportune to reexamine the global strategy.

Global estimates of malaria burden are dominated by infections due to *Plasmodium falciparum* in sub-Saharan Africa. The recent frameworks prioritize the challenges to overcome this particular malaria problem, but downplay the significance of the other five parasite species causing human malaria, which dominate the epidemiology in other regions. If global eradication of human malaria is the goal, then compared with *P. falciparum*, these other pathogens pose problems of greater complexity, geographic range, and technical difficulty, with fewer tools available to tackle them and more unknowns. The major challenges that arise on the pathway to malaria eradication have been stated before, by the then director of the Global Malaria Eradication Campaign:

*…..but it was thought useful to draw attention […] to the commonplace truth that if malaria eradication is technically possible, it is and will always remain a very serious task, and that governments should not embark on it underestimating its difficulties, or hoping that as the years pass some of the problems will automatically be cleared *….*^4^* *(Emilio Pampana, 1958)*

*Plasmodium vivax* in particular is highly prevalent across endemic Asia and South America but also occurs in Africa.^5^ Vivax malaria is associated with severe morbidity and with mortality, but our current clinical and public health tools for its diagnosis, prevention, treatment, and control are suboptimal in many endemic areas.^6^ Malaria due to *Plasmodium malariae* is present in most malaria-endemic settings and associated with greatly prolonged parasite carriage, severe anemia and renal impairment. In several regions in Asia, zoonotic knowlesi malaria is now the predominant cause of clinical and severe malaria.^7^ Similar to *P. vivax*, the two sympatric species of *Plasmodium ovale* produce dormant liver stages that can relapse months after the initial infection and are also associated with severe and life-threatening syndromes.

Eliminating malaria one species at a time may increase the time and resources required. Rather than initially targeting the species of greatest ease to eliminate, a strategy targeting all species in parallel may be the more efficient and successful strategy. A focus on all-species elimination should not come at the expense of progress in reducing global mortality from falciparum malaria, but eradication is not just simply minimizing burden, and moreover, all species cause morbidity and mortality.^8^ Conversely, if the ultimate aim is indeed limited to eliminating the most harmful species, then the global community must reformulate and express this goal in more specific terms.

## THE CHALLENGE OF THE FINAL STAGES

Eradication is a biological process whereby a living entity no longer exists in the natural world as a consequence of sustained and deliberate human interventions.^9^ The simple phrase “malaria eradication” obscures the complex reality of all six species of *Plasmodium* being driven to extinction. For a single one (*P. falciparum*), a well-defined path has been charted and for a second (*P. vivax)*, a strategic plan is rapidly evolving,^2^ but there are no technical agendas for *P. malariae*, either *P. ovale* subspecies or for *Plasmodium knowlesi*. It is recognized that current tools are inadequate for elimination^10^ and until now, little thought and attention has been given toward *P. malariae*, *P. ovale* sp., or *P. knowlesi*, and understanding of the biology and transmission dynamics of each of these species will be required. Moreover, the burden of morbidity and mortality from these species is poorly understood and almost certainly underestimated.^7,11,12^

All disease eradication programs have encountered surprising complexities in the final stages. As Bruce-Chwatt suggested decades ago, “Malaria eradication has been compared with high-mountain climbing: more obstacles appear as one approaches the summit, and at that stage every step demands an increased effort.”^13^ Examples of challenges in the end game include the identification of human monkey pox infections in the final phases of the smallpox campaign, the recognition of sylvatic reservoirs of yellow fever virus, and the recent detection of canine hosts and potential aquatic reservoirs of Guinea worm.^14,15^ Furthermore, nearly all elimination efforts have been directed toward a *single species* of pathogen and most had the advantage of a vaccine providing sterilizing immunity, including polio and measles. Malaria raises issues of far greater complexity as it involves at least 10 intermediate host/reservoir species globally, and more than 40 major anopheline vector species. Diverse systems may need to be considered to achieve total elimination and subsequent eradication of the malarias from humanity—a one-size approach certainly will not fit all.

Four broad and interconnected ideas frame the specific scientific issues that need to be addressed for malaria elimination: diversities within a single parasite species, zoonotic and anthroponotic parasite transfers, the diverse bioecology of each parasite species, and the extensive diversities within both human hosts and vectors. Strategies for elimination and eradication that do not incorporate and address these complexities may lead to delays and even failure as each of these directly impact the themes described in the following paragraphs.

## THE SHIFTING BURDEN OF DISEASE

Recent malaria control efforts have reduced significantly the global burden of malaria,^16^ yet in many regions where *P. falciparum* and other *Plasmodium* are co-endemic, there has been a relative increase in the proportion of infections due to the five other species, especially where PCR-based testing is performed.^3,17^ *Plasmodium vivax* is estimated to cause approximately 14 million clinical episodes of malaria each year,^12^ but much larger estimates also exist.^18^ Once considered a benign infection, vivax malaria is now recognized to cause severe and fatal outcomes across a range of epidemiological settings.^19^ This parasite also causes extensive indirect mortality attributable to recurrent infections and a cumulative risk of severe anemia.^20^ Outside sub-Saharan Africa, *P. vivax* is now often the predominant cause of malaria; however, this shift in the burden of disease has yet to be appropriately weighted in research prioritization.

In Malaysia, *P. knowlesi* is the leading cause of malaria morbidity and mortality.^7^ *Plasmodium malariae* contributes significant morbidity in sub-Saharan Africa and elsewhere.^21^ In areas of stable malaria transmission, almost 10% of clinical malaria can be attributed to *P. malariae* and its ability to maintain prolonged low-level parasitemias is associated with a high burden of anemia and hospitalization,^22,23^ tropical splenomegaly,^24^ and with the potential for irreversible kidney damage.^25^ The two sympatric species of *P. ovale* have differing epidemiology^26^ and are often misidentified as other species in the clinic by microscopy, suggesting underreporting. Finally, all five of these species, including *P. knowlesi*, have been associated with fatal outcomes in well-resourced settings.^8,27^ The common distinction between harmful and harmless *Plasmodium* species has been discredited by available evidence. Priority assigned to *P. falciparum* on this basis dangerously minimizes the clinical and public health importance of the other *Plasmodium* species.

## SYLVATIC MALARIAS

Over the past decade, compelling evidence has revealed continual interactions between malaria species in human and nonhuman primate reservoirs, driven largely by the discovery of widespread *P. knowlesi* cases in Southeast Asia.^28^ Several zoonotic reservoirs of malaria have been confirmed,^29^ and most recently, a common parasite of howler monkeys *Plasmodium simium*,^30^ was found to have regular spillover into humans with initial misidentification as *P. vivax* in Brazil,^31^ from which it is nearly indistinguishable.^32^ Genetic evidence also suggests regular exchange of *P. malariae* between humans and nonhuman primates in the Venezuelan Amazon.^33^ Finally, possibly anthroponotic *P. malariae*, *P. ovale*, and *P. vivax* have been found in wild “pristine” nonhuman primate populations in Central Africa.^34^

Major challenges exist in studying parasites in these primate reservoirs, and currently, there are limited tools or sampling strategies to estimate the frequency of parasite transfer between humans and primates in these populations. This lack of evidence for a stable animal reservoir of human malarias cannot be considered as evidence of absence. The geographic extents and magnitude of animal-associated malaria in humans (whether zoonotic and anthroponotic) need to be quantified to ensure that malaria eradication is an evidence-based undertaking,^9^ a challenge identified during the Global Malaria Eradication era, which remains unaddressed.^35,36^ The potential burden of disease attributable to the transfer of parasites between humans and primate reservoirs is unknown, the assumption that this is negligible needs to be explored further to ensure evidence-based eradication programs.

## BIOLOGY OF THE PARASITES

*Plasmodium vivax* has evolved an ability to sustain transmission in environments inhospitable to *P. falciparum*. Central to this survival strategy is the dormant liver stage (hypnozoite) capable of relapsing weeks, months, and even several years after the initial mosquito-borne infection. Furthermore, this species can persist with much lower peripheral parasitemias, with all stages of the parasite, including sexual stages (gametocytes—the parasite life stage infectious to mosquitoes), circulating before patients developing symptoms.

Gametocytes of *P. vivax* are able to transmit to a larger range of vectors and complete the parasite stages in the mosquito over a much wider range of temperatures than *P. falciparum*.^37^ Conversely, the sexual stages in *P. falciparum* infection tend to occur days to weeks after clinical presentation, allowing transmission-blocking interventions such as administration of a single dose of primaquine (PQ).^38^ Unlike the gametocytes of *P. falciparum*, those of *P. vivax* are inherently sensitive to most blood schizontocidal treatments and do not persist for any significant length of time following clearance of asexual parasitemia.

The main driving force of *P. vivax* transmission is its ability to relapse, a property that accounts for most of the subsequent recurrent infections following an initial infection.^39,40^ Hence, good coverage of *P. vivax* radical cure is likely to reduce transmission to a far greater extent than strategies focused solely on treatment of symptomatic patients.^41^ Numerous studies have shown that similar investments in malaria control measures have a large differential impact on the incidence of *P. falciparum* relative to *P. vivax*.^42^ In some instances, hotspots of *P. vivax* transmission may persist for years with a significant risk of resurgence.^43–45^ The primary reason for this is the inability to deliver safe, effective, and acceptable radical cure of the hypnozoite reservoir due to the risk of PQ-induced hemolysis in glucose 6-phosphate dehydrogenase (G6PD)–deficient patients along with the necessity of prolonged dosing (2 weeks) and attendant barriers to adherence.

Finally, although generally regarded as not posing important barriers to elimination, the highly complex and currently obscure biology and epidemiology of both species *P. ovale* and *P. malariae*, especially concerning quiescent parasite stages, should not be ignored.^26,46^

## SPECIES-SPECIFIC INTERACTIONS WITH ANOPHELINE VECTORS

A wider range of vectors is capable of transmitting *P. vivax* relative to *P. falciparum*,^47^ with two important consequences. First, transmission can persist in areas with multiple potential vectors after control of the primary vector species. Second, persistent foci may be highly resilient in spite of changing ecological conditions, because of both changes in vector composition (e.g., Kenya^48^ and Amazonia^49^) and in biting behavior (e.g., Solomon Islands^42^).

Many of the vector complexes outside of sub-Saharan Africa are less impacted by insecticide-treated nets because of exophagy and exophily,^50^ and the evidence base for many key entomological interventions outside of sub-Saharan Africa (especially in *P. vivax*–endemic regions) is remarkably sparse.^51–53^ In addition, proposed interventions for areas with exophilic vectors have had limited or no impact at scale, including treated hammocks^54^ and topical repellants.^55^

Vector populations in sub-Saharan Africa hide unexpected complexity,^56^ and many cryptic or presumed secondary vectors may in fact be major contributors to transmission.^57,58^ The presence of urban vectors (including *Anopheles stephensi* on the Indian subcontinent and the Horn of Africa^59^), and adaptation of *Anopheles* spp. larvae to polluted water both represent another potentially important barrier to elimination. Regular large-scale field surveys and concurrent laboratory studies will be required to ensure that these adaptive malaria vectors do not become both globally dispersed or resistant to interventions as *Aedes* spp. have become in many dengue-endemic areas.^60,61^

The efforts required to identify cryptic vector populations in residual transmission is highlighted by work in Borneo during the 1940s.^62^ While field work captured ample female *Anopheles,* the sporozoite rates of all suspected vectors were too low to support the observed malaria prevalence, and 2 years of dedicated study was required to identify miniscule jungle breeding sites for *Anopheles leucosphyrus*.^62^ Rationally targeting residual transmission may require similar levels of intensive entomology to define individual disease ecotypes.^63^ For subsequent targeting of these niches, housing improvements and the generally neglected, historical interventions focused around environmental modifications need be closely reexamined.^64–66^

## REDUCING TRANSMISSION BY TARGETING THE PARASITE RESERVOIRS

The proportion of all parasites in the human reservoir that are subpatent is far greater in *P. vivax* than in *P. falciparum,* because of inherently lower parasitemias, the relative insensitivity of both microscopy and rapid diagnostic tests, and the presence of undetectable parasites in the liver, bone marrow, and spleen.^67^ Recent studies suggest that parasite densities are approximately 5-fold lower in *P. vivax* infections than in *P. falciparum* infections,^68^ although the exact magnitude varies with methodology.^69,70^ This problem is compounded by the species’ transmissibility to mosquito vectors at very low levels of parasitemia, greatly increasing the size of the infectious reservoir relative to *P. falciparum*.^71^

Greater genetic diversity of global parasite populations highlights the need for interventions to be tailored to specific epidemiological settings, particularly with respect to relapse patterns and incubation periods.^72,73^ Even in very low-transmission settings, *P. vivax* genetic diversity remains significantly higher than that of *P. falciparum*, a likely reflection of the underlying contribution of hypnozoites,^74,75^ and such intrinsic genetic diversity provide the parasite with an increased ability to respond to challenges to its survival.^76^

## HOST SUSCEPTIBILITY

The impact of host genetics on risk of malaria infection is poorly understood, although there are important differences in host susceptibility to infection.^77^ The genetic diversity of host populations across *P. vivax*–endemic areas is extensive (including Central and South America, northern and eastern Africa, southern and southeastern Asia, and much of Oceania). Evidence suggests that *P. vivax* may be rapidly evolving alternate invasion pathways.^78^ Parasite populations have been identified capable of “invading” Duffy-negative human populations along with increasing virulence^79,80^ and genomic studies suggests that these non-Duffy antigen invasion pathways appeared very recently. Identification of novel invasion pathways in Duffy-negative individuals suggest the potential for expansion of populations to be at risk from *P. vivax* to include much of sub-Saharan Africa.^81^

## THERAPEUTIC REGIMENS

Chloroquine remains the first-line treatment of *P. vivax* in most endemic areas because of its widespread availability, sustained efficacy, low cost, and excellent safety and tolerability profile. However, resistant strains have been identified across the vivax-endemic world, especially in Southeast Asia.^82^ Given widespread resistance to chloroquine and difficulties of malaria species identification, several countries have changed national policy to ACTs for all malaria infections (including Indonesia, Malaysia, Cambodia, Papua New Guinea, the Solomon Islands, and Vanuatu).^83,84^ Although both *P. ovale* and *P. malariae* are assumed to be sensitive to chloroquine, data on drug susceptibility of these species are lacking.^85^ The dearth of research into these issues is highlighted by the total number of registered trials currently listed as “recruiting” or “completed” by species: “radical cure” for vivax (15 trials) or ovale (one); “treatment” for malariae (four), and “treatment” for knowlesi (four trials).^86^

Primaquine, an 8-aminoquinoline, is the only currently available hypnozoiticide but causes severe drug-induced hemolysis in G6PD-deficient patients. To improve safety and tolerability, PQ is usually administered as a 14-day regimen, but this is associated with poor adherence to a prolonged course of treatment with consequent poor effectiveness.^87–89^ Further complexities also exist in the use of PQ. There are wide variations in prevalence and severity of G6PDd, which typically range from 1% to 30% (mean 8%) in malaria-endemic regions.^90^ Glucose 6-phosphate dehydrogenase deficiency in males are usually associated with an enzyme activity of less than 30% of normal levels, with some variants being 10% or lower. These “severe” (low activity) variants are highly susceptible to PQ-associated hemolytic toxicity, which may require immediate clinical management, including blood transfusion. Primaquine is also contraindicated in pregnant women as the G6PD status of the fetus is unknown, and in infants less than 6 months of age. However, these exact groups are the most vulnerable to the serious adverse health effects of recurrent vivax malaria.

The WHO recommends that all *P. vivax* patients be tested for G6PD deficiency before treatment with PQ; however, testing is not standard-of-care in most settings. Although rapid tests for G6PD deficiency have been developed, expansion into routine use will require considerable effort and finances. Furthermore, most qualitative G6PD assays usually return a normal result for enzyme activity greater than 30% normal, missing heterozygous females, who may have mixtures of G6PD normal and deficient red blood cells and are therefore at risk of hemolysis.

Hypnozoite carriage is asymptomatic and there are currently no diagnostics able to detect their presence—hence the only reliable way to attempt to clear out the hypnozoite reservoir in populations is through presumptive treatment to those at risk and/or mass drug administration (MDA). Mass drug administration using PQ for radical cure has been implemented at a massive scale, giving doses to millions of people in the former Soviet Republics, People’s Republic of China, and the Democratic Republic of Korea (North Korea); but differences exist in reporting and G6PDd prevalence.^91^ More recently, an MDA campaign including PQ radical cure in Greece achieved good coverage (76%) in ≈1,200 people, but required enormous resources, and one patient required hospitalization for PQ-induced hemolysis after a false-normal *laboratory-based* G6PD test.^92^ Although high compliance can be achieved, it is a major and expensive undertaking—the Greek endeavor required multiple teams making regular household visits for all enrolled persons.^92^

The efficacy of PQ varies by parasite populations, which may require different dosing to prevent relapses,^93^ and the relative risk-benefit of PQ radical cure may vary in different epidemiological settings.^94^ The biological activity of PQ against hypnozoites may require the formation of reactive intermediates generated by the cytochrome P-450 isozyme 2D6 (CYP2D6). Recent clinical trials identified a spectrum of clinical responses to PQ based on the host’s CYP2D6 isoform ranging from null- to ultra-metabolizers.^95^ These polymorphisms are widely variable among ethnic groups, but there are currently no population-level data to assess the potential impact on PQ efficacy. One study in Indonesia found that 20 of 21 PQ treatment failures among 171 patients were indeed associated with impaired CYP2D6 genotypes and phenotypes (J. K. Baird, personal communication). Interactions between parasite and host genetics may also play a role.

In summary, PQ is the only hypnozoitocidal drug now available; it has the potential for severe and fatal adverse events, major issues in patient adherence, and is contraindicated in the most vulnerable populations in need of radical cure. Hence, a safe and effective therapy for the radical cure of vivax malaria is needed. A drug currently in Phase III trials, tafenoquine, may simplify adherence, but this will require routine testing for G6PD deficiency.

## CONCLUSION

The diversity and complexity of the human malarias are great, presenting huge challenges to the ambitious goal of eradicating malaria by 2040. The tools currently available have been focused on *P. falciparum*, focused on reducing the high burdens of this parasite and its associated morbidity and mortality. However, in both a practical and technical sense, none of these tools are ideally suited to either the distinct task of bringing low-level transmission to zero or dealing with all of the *Plasmodium* parasites and their anopheline vectors. Because all malaria species in humans contribute severe morbidity and mortality, a roadmap attacking the easiest target species first and then moving on to the next needs to be reexamined. A strategy that addresses the combination of *Plasmodium* species where they exist will decrease the time, resources, and energy required to achieve global malaria eradication.

A focus on all species of *Plasmodium* should not come at the expense of progress in reducing global mortality from falciparum malaria; however, the goal of malaria eradication does not equate to simply minimizing disease burden. As all species can cause morbidity and mortality,^8^ malaria eradication must by definition encompass them all. However, if the ultimate goal is indeed limited to eliminating the most harmful species, then the global community must reformulate and express this goal in more specific terms. A more comprehensive research agenda is required to ensure continued progress (Table 1). The current eradication environment is built on optimism and a belief that the ecological or epidemiological subtleties will not impede our progress. We are therefore at an opportune moment where we can embrace these barriers and integrate their solutions into the new global eradication program.

**Table 1:** A proactive agenda to promote elimination of all species of human malaria

1. Surveillance of *Plasmodium vivax* in new populations and expansion of the *P. vivax* elimination agenda to include sub-Saharan Africa
2. Geospatial mapping of glucose 6-phosphate dehydrogenase deficiencies and CYP2D6 to gauge populations at risk of primaquine-induced hemolysis and poor efficacy
3. Optimizing drug treatment of *P. vivax*, *Plasmodium ovale*, *Plasmodium malariae*, and *Plasmodium knowlesi*
4. Ensure effective radical cure of malaria through improved adherence or short-duration treatment regimens
5. Reestablish field entomology as a core component of malariology
6. Incorporation of ecology (including environmental control methods) into malaria planning, where appropriate
7. Increase sampling of parasite reservoirs in areas with potential zoonotic transmission (human and nonhuman primate) and phylogeny of parasite populations to asses “spillover”
8. Identification and optimization of strategies to target zoonotic malaria infections
9. Greater emphasis on the basic biology of *P. knowlesi*, *P. ovale*, and *P. malariae*

## References

[1] Gates B Chambers R, 2015 *From Aspiration to Action* Available at: http://endmalaria2040.org/assets/Aspiration-to-Action.pdf. Accessed November 19, 2015.

[2] World Health Organization, 2015 *Control and Elimination of Plasmodium vivax Malaria: A Technical Brief* Geneva, Switzerland: WHO. Available at: http://www.who.int/iris/handle/10665/181162. Accessed September 8, 2016.

[3] World Health Organization, 2017 *World Malaria Report 2017*. Geneva, Switzerland: Global Malaria Program, WHO.

[4] Pampana EJ, 1958 *Unexpected Cost Increases of Malaria Eradication Programmes* Geneva, Switzerland: World Health Organization. Available at: http://www.who.int/iris/handle/10665/64589. Accessed August 25, 2017.

[5] Howes RE 2015 *Plasmodium vivax* transmission in Africa. PLoS Negl Trop Dis 9: e0004222. 26587988 10.1371/journal.pntd.0004222 PMC4654493

[6] Mueller I Galinski MR Baird JK Carlton JM Kochar DK Alonso PL del Portillo HA, 2009 Key gaps in the knowledge of *Plasmodium vivax*, a neglected human malaria parasite. Lancet Infect Dis 9: 555–566. 19695492 10.1016/S1473-3099(09)70177-X

[7] Barber BE William T Grigg MJ Menon J Auburn S Marfurt J Anstey NM Yeo TW, 2013 A prospective comparative study of knowlesi, falciparum, and vivax malaria in Sabah, Malaysia: high proportion with severe disease from *Plasmodium knowlesi* and *Plasmodium vivax* but no mortality with early referral and artesunate therapy. Clin Infect Dis 56: 383–397. 23087389 10.1093/cid/cis902

[8] Hwang J Cullen K Kachur SP Arguin PM Baird JK, 2014 Severe morbidity and mortality risk from malaria in the United States, 1985–2011. Open Forum Infect Dis 1: ofu034. 25734104 10.1093/ofid/ofu034 PMC4324198

[9] Dowdle WR Hopkins D, 1998 *The Eradication of Infectious Diseases*, Vol. 24. Chichester, United Kingdom: John Wiley & Sons.

[10] Tanner M 2015 Malaria eradication and elimination: views on how to translate a vision into reality. BMC Med 13: 167. 26208740 10.1186/s12916-015-0384-6 PMC4514994

[11] Roucher C Rogier C Sokhna C Tall A Trape J-F, 2014 A 20-year longitudinal study of *Plasmodium ovale* and *Plasmodium malariae* prevalence and morbidity in a West African population. PLoS One 9: e87169. 24520325 10.1371/journal.pone.0087169 PMC3919715

[12] Howes RE Battle KE Mendis KN Smith DL Cibulskis RE Baird JK Hay SI, 2016 Global epidemiology of *Plasmodium vivax*. Am J Trop Med Hyg 95 (*6 Suppl*): 15–34. 10.4269/ajtmh.16-0141 PMC5198891 27402513

[13] Bruce-Chwatt LJ, 1965 Malaria research for malaria eradication. Trans R Soc Trop Med Hyg 59: 105–144. 14297189 10.1016/0035-9203(65)90073-8

[14] Arita I Jezek Z Khodakevich L Ruti K, 1985 Human monkeypox: a newly emerged orthopoxvirus zoonosis in the tropical rain forests of Africa. Am J Trop Med Hyg 34: 781–789. 2992305 10.4269/ajtmh.1985.34.781

[15] Eberhard ML 2014 The peculiar epidemiology of dracunculiasis in Chad. Am J Trop Med Hyg 90: 61–70. 24277785 10.4269/ajtmh.13-0554 PMC3886430

[16] World Health Organization, 2015 *World Malaria Report 2015* Geneva, Switzerland: WHO. Available at: http://www.who.int/malaria/publications/world-malaria-report-2015/report/en/. Accessed December 9, 2015.

[17] Sattabongkot J Tsuboi T Zollner GE Sirichaisinthop J Cui L, 2004 *Plasmodium vivax* transmission: chances for control? Trends Parasitol 20: 192–198. 15099559 10.1016/j.pt.2004.02.001

[18] Price RN Tjitra E Guerra CA Yeung S White NJ Anstey NM, 2007 Vivax malaria: neglected and not benign. Am J Trop Med Hyg 77 (*6 Suppl*): 79–87. 18165478 PMC2653940

[19] Anstey NM Douglas NM Poespoprodjo JR Price RN, 2012 *Plasmodium vivax*: clinical spectrum, risk factors and pathogenesis. Hay SI, Price RN, Baird JK, eds. *Advances in Parasitology*, Vol. 80. Oxford, United Kingdom: Academic Press, 151–201. Available at: http://www.sciencedirect.com/science/article/pii/B9780123979001000037. Accessed September 28, 2014. 10.1016/B978-0-12-397900-1.00003-7 23199488

[20] Price RN Douglas NM Anstey NM, 2009 New developments in *Plasmodium vivax* malaria: severe disease and the rise of chloroquine resistance. Curr Opin Infect Dis 22: 430–435. 19571748 10.1097/QCO.0b013e32832f14c1

[21] Camargo-Ayala PA Cubides JR Niño CH Camargo M Rodríguez-Celis CA Quiñones T Sánchez-Suárez L Patarroyo ME Patarroyo MA, 2016 High *Plasmodium malariae* prevalence in an endemic area of the Colombian Amazon region. PLoS One 11: e0159968. 27467587 10.1371/journal.pone.0159968 PMC4965042

[22] Douglas NM Lampah DA Kenangalem E Simpson JA Poespoprodjo JR Sugiarto P Anstey NM Price RN, 2013 Major burden of severe anemia from non-falciparum malaria species in southern Papua: a hospital-based surveillance study. PLoS Med 10: e1001575. 24358031 10.1371/journal.pmed.1001575 PMC3866090

[23] Langford S Douglas NM Lampah DA Simpson JA Kenangalem E Sugiarto P Anstey NM Poespoprodjo JR Price RN, 2015 *Plasmodium malariae* infection associated with a high burden of anemia: a hospital-based surveillance study. PLoS Negl Trop Dis 9: e0004195. 26720002 10.1371/journal.pntd.0004195 PMC4697806

[24] Leoni S Buonfrate D Angheben A Gobbi F Bisoffi Z, 2015 The hyper-reactive malarial splenomegaly: a systematic review of the literature. Malar J 14: 185. 25925423 10.1186/s12936-015-0694-3 PMC4438638

[25] Collins WE Jeffery GM, 2007 *Plasmodium malariae*: parasite and disease. Clin Microbiol Rev 20: 579–592. 17934075 10.1128/CMR.00027-07 PMC2176047

[26] Sutherland CJ, 2016 Persistent parasitism: the adaptive biology of malariae and ovale malaria. Trends Parasitol 32: 808–819. 27480365 10.1016/j.pt.2016.07.001

[27] Cox-Singh J 2010 Severe malaria—a case of fatal *Plasmodium knowlesi* infection with post-mortem findings: a case report. Malar J 9: 10. 20064229 10.1186/1475-2875-9-10 PMC2818646

[28] Singh B Kim Sung L Matusop A Radhakrishnan A Shamsul SSG Cox-Singh J Thomas A Conway DJ, 2004 A large focus of naturally acquired *Plasmodium knowlesi* infections in human beings. Lancet 363: 1017–1024. 15051281 10.1016/S0140-6736(04)15836-4

[29] Prugnolle F 2013 Diversity, host switching and evolution of *Plasmodium vivax* infecting African great apes. Proc Natl Acad Sci USA 110: 8123–8128. 23637341 10.1073/pnas.1306004110 PMC3657773

[30] Costa DC da Cunha VP de Assis GMP de Souza Junior JC Hirano ZMB de Arruda ME Kano FS Carvalho LH de Brito CFA, 2014 *Plasmodium simium/Plasmodium vivax* infections in southern brown howler monkeys from the Atlantic Forest. Mem Inst Oswaldo Cruz 109: 641–653. 25099335 10.1590/0074-0276130578 PMC4156457

[31] Brasil P 2017 Outbreak of human malaria caused by *Plasmodium simium* in the Atlantic Forest in Rio de Janeiro: a molecular epidemiological investigation. Lancet Glob Health 5: e1038–e1046. 28867401 10.1016/S2214-109X(17)30333-9

[32] Lim CS Tazi L Ayala FJ, 2005 *Plasmodium vivax*: recent world expansion and genetic identity to *Plasmodium simium*. Proc Natl Acad Sci USA 102: 15523–15528. 16227436 10.1073/pnas.0507413102 PMC1266129

[33] Lalremruata A Magris M Vivas-Martínez S Koehler M Esen M Kempaiah P Jeyaraj S Perkins DJ Mordmüller B Metzger WG, 2015 Natural infection of *Plasmodium brasilianum* in humans: man and monkey share quartan malaria parasites in the Venezuelan Amazon. EBioMedicine 2: 1186–1192. 26501116 10.1016/j.ebiom.2015.07.033 PMC4588399

[34] Kaiser M 2010 Wild chimpanzees infected with 5 *Plasmodium* species. Emerg Infect Dis 16: 1956–1959. 21122230 10.3201/eid1612.100424 PMC3294549

[35] Bruce-Chwatt LJ, 1968 Malaria zoonosis in relation to malaria eradication. Trop Geogr Med 20: 50–87. 4966749

[36] Contacos PG, 1970 Primate malarias: man and monkeys. J Wildl Dis 6: 323–328. 16512134 10.7589/0090-3558-6.4.323

[37] World Health Organization, Global Malaria Programme, 2007 *Malaria Elimination: A Field Manual for Low and Moderate Endemic Countries* Geneva, Switzerland: WHO. Available at: http://apps.who.int//iris/handle/10665/43796. Accessed November 13, 2014.

[38] Steketee RW ter Kuile F, 2013 Single low-dose primaquine to reduce malaria transmission. Lancet Infect Dis 14: 91–92. 24239326 10.1016/S1473-3099(13)70288-3

[39] White MT Karl S Battle KE Hay SI Mueller I Ghani AC, 2014 Modelling the contribution of the hypnozoite reservoir to *Plasmodium vivax* transmission. eLife 3: e04692. 10.7554/eLife.04692 PMC4270097 25406065

[40] Robinson LJ 2015 Strategies for understanding and reducing the *Plasmodium vivax* and *Plasmodium ovale* hypnozoite reservoir in Papua New Guinean children: a randomised placebo-controlled trial and mathematical model. PLoS Med 12: e1001891. 26505753 10.1371/journal.pmed.1001891 PMC4624431

[41] Douglas NM John GK von Seidlein L Anstey NM Price RN, 2012 Chemotherapeutic strategies for reducing transmission of *Plasmodium vivax* malaria. Adv Parasitol 80: 271–300. 23199490 10.1016/B978-0-12-397900-1.00005-0

[42] Waltmann A 2015 High rates of asymptomatic, sub-microscopic *Plasmodium vivax* infection and disappearing *Plasmodium falciparum* malaria in an area of low transmission in Solomon Islands. PLoS Negl Trop Dis 9: e0003758. 25996619 10.1371/journal.pntd.0003758 PMC4440702

[43] Kaneko A 2014 Characteristic age distribution of *Plasmodium vivax* infections after malaria elimination on Aneityum Island, Vanuatu. Infect Immun 82: 243–252. 24166950 10.1128/IAI.00931-13 PMC3911855

[44] Danis K Baka A Lenglet A Van Bortel W Terzaki I Tseroni M Detsis M Papanikolaou E Balaska A Gewehr S, 2011 Autochthonous *Plasmodium vivax* malaria in Greece, 2011. Euro Surveill 16: 20. 22027375

[45] Iwagami M Hwang S-Y Kim S-H Park S-J Lee G-Y Matsumoto-Takahashi ELA Kho W-G Kano S, 2013 Microsatellite DNA analysis revealed a drastic genetic change of *Plasmodium vivax* population in the Republic of Korea during 2002 and 2003. PLoS Negl Trop Dis 7: e2522. 24205429 10.1371/journal.pntd.0002522 PMC3814342

[46] Markus MB, 2017 Malaria eradication and the hidden parasite reservoir. Trends Parasitol 33: 492–495. 28366603 10.1016/j.pt.2017.03.002

[47] Daskova NG Rasnicyn SP, 1982 Review of data on susceptibility of mosquitos in the USSR to imported strains of malaria parasites. Bull World Health Organ 60: 893–897. 6761003 PMC2535980

[48] Bayoh MN Mathias DK Odiere MR Mutuku FM Kamau L Gimnig JE Vulule JM Hawley WA Hamel MJ Walker ED, 2010 *Anopheles gambiae*: historical population decline associated with regional distribution of insecticide-treated bed nets in western Nyanza Province, Kenya. Malar J 9: 62. 20187956 10.1186/1475-2875-9-62 PMC2838909

[49] da Silva-Nunes M Moreno M Conn JE Gamboa D Abeles S Vinetz JM Ferreira MU, 2012 Amazonian malaria: asymptomatic human reservoirs, diagnostic challenges, environmentally driven changes in mosquito vector populations, and the mandate for sustainable control strategies. Acta Trop 121: 281–291. 22015425 10.1016/j.actatropica.2011.10.001 PMC3308722

[50] Smithuis 2013 Entomological determinants of insecticide-treated bed net effectiveness in western Myanmar. Malar J 12: 364. 24119994 10.1186/1475-2875-12-364 PMC4015723

[51] Dolan G Ter Kuile FO Jacoutot V White NJ Luxemburger C Malankirii L Chongsuphajaisiddhi T Nosten F, 1993 Bed nets for the prevention of malaria and anaemia in pregnancy. Trans R Soc Trop Med Hyg 87: 620–626. 8296357 10.1016/0035-9203(93)90262-o

[52] Lengeler C, 2004 Insecticide-treated bed nets and curtains for preventing malaria. Cochrane Database Syst Rev 2: CD000363. 10.1002/14651858.CD000363.pub2 15106149

[53] Smithuis FM 2013 The effect of insecticide-treated bed nets on the incidence and prevalence of malaria in children in an area of unstable seasonal transmission in western Myanmar. Malar J 12: 1. 24119916 10.1186/1475-2875-12-363 PMC3854704

[54] Grietens KP Nguyen Xuan X Muela Ribera J Ngo Duc T van Bortel W Truong Ba N Van KP Le Xuan H D’Alessandro U Erhart A, 2012 Social determinants of long lasting insecticidal hammock-use among the Ra-glai ethnic minority in Vietnam: implications for forest malaria control. PLoS One 7: e29991. 22253852 10.1371/journal.pone.0029991 PMC3257264

[55] Chen-Hussey V Carneiro I Keomanila H Gray R Bannavong S Phanalasy S Lindsay SW, 2013 Can topical insect repellents reduce malaria? A cluster-randomised controlled trial of the insect repellent *n,n*-diethyl-*m*-toluamide (DEET) in Lao PDR. PLoS One 8: e70664. 23967083 10.1371/journal.pone.0070664 PMC3743820

[56] Lobo NF 2016 Unexpected diversity of *Anopheles* species in eastern Zambia: implications for evaluating vector behavior and interventions using molecular tools. Sci Rep 5: 17952. 10.1038/srep17952 PMC4673690 26648001

[57] Stevenson J St. Laurent B Lobo NF Cooke MK Kahindi SC Oriango RM Harbach RE Cox J Drakeley C, 2012 Novel vectors of malaria parasite in the Western Highlands of Kenya. Emerg Infect Dis 18: 1547–1549. 22932762 10.3201/eid1809.120283 PMC3437730

[58] Stevenson JC Norris DE, 2016 Implicating cryptic and novel Anophelines as malaria vectors in Africa. Insects 8: 1. 10.3390/insects8010001 PMC5371929 28025486

[59] Faulde MK Rueda LM Khaireh BA, 2014 First record of the Asian malaria vector *Anopheles stephensi* and its possible role in the resurgence of malaria in Djibouti, Horn of Africa. Acta Trop 139: 39–43. 25004439 10.1016/j.actatropica.2014.06.016

[60] Gunathilaka N Fernando T Hapugoda M Wickremasinghe R Wijeyerathne P Abeyewickreme W, 2013 *Anopheles culicifacies* breeding in polluted water bodies in Trincomalee district of Sri Lanka. Malar J 12: 1. 23958454 10.1186/1475-2875-12-285 PMC3765073

[61] Ramasamy R Surendran SN, 2016 Mosquito vectors developing in atypical anthropogenic habitats: global overview of recent observations, mechanisms and impact on disease transmission. J Vector Borne Dis 53: 91. 27353577

[62] McArthur J, 1947 The transmission of malaria in Borneo. Trans R Soc Trop Med Hyg 40: 537–558. 20243878 10.1016/0035-9203(47)90020-5

[63] Schapira A Boutsika K, 2012 Malaria ecotypes and stratification. *Advances in Parasitology*, Rollinson D, Hay SI, eds. Vol. 78. London: Academic Press, 97–167. Available at: http://linkinghub.elsevier.com/retrieve/pii/B9780123943033000013. Accessed September 12, 2012. 10.1016/B978-0-12-394303-3.00001-3 22520442

[64] Tusting LS Ippolito MM Willey BA Kleinschmidt I Dorsey G Gosling RD Lindsay SW, 2015 The evidence for improving housing to reduce malaria: a systematic review and meta-analysis. Malar J 14: 209. 26055986 10.1186/s12936-015-0724-1 PMC4460721

[65] Boyd MF, 1949 *Malariology: A Comprehensive Survey of All Aspects of This Group of Diseases from a Global Standpoint*, 1st edition. Philadelphia and London: Saunders.

[66] Keiser J Singer BH Utzinger J, 2005 Reducing the burden of malaria in different eco-epidemiological settings with environmental management: a systematic review. Lancet Infect Dis 5: 695–708. 16253887 10.1016/S1473-3099(05)70268-1

[67] Chen I Clarke SE Gosling R Hamainza B Killeen G Magill A O’Meara W Price RN Riley EM, 2016 “Asymptomatic” malaria: a chronic and debilitating infection that should be treated. PLoS Med 13: e1001942. 26783752 10.1371/journal.pmed.1001942 PMC4718522

[68] Koepfli C 2015 Blood-stage parasitaemia and age determine *Plasmodium falciparum* and *P. vivax* gametocytaemia in Papua New Guinea. PLoS One 10: e0126747. 25996916 10.1371/journal.pone.0126747 PMC4440770

[69] Moreira CM Abo-Shehada M Price RN Drakeley CJ, 2015 A systematic review of sub-microscopic *Plasmodium vivax* infection. Malar J 14: 360. 26390924 10.1186/s12936-015-0884-z PMC4578340

[70] Imwong M 2016 Numerical distributions of parasite densities during asymptomatic malaria. J Infect Dis 213: 1322–1329. 26681777 10.1093/infdis/jiv596 PMC4799672

[71] Gamage-Mendis AC Rajakaruna J Carter R Mendis KN, 1991 Infectious reservoir of *Plasmodium vivax* and *Plasmodium falciparum* malaria in an endemic region of Sri Lanka. Am J Trop Med Hyg 45: 479–487. 1951856 10.4269/ajtmh.1991.45.479

[72] Lover AA Coker RJ, 2013 Quantifying effect of geographic location on epidemiology of *Plasmodium vivax* malaria. Emerg Infect Dis 19: 1058–1065. 23763820 10.3201/eid1907.121674 PMC3713979

[73] Battle KE 2014 Geographical variation in *Plasmodium vivax* relapse. Malar J 13: 144. 24731298 10.1186/1475-2875-13-144 PMC4021508

[74] Gunawardena S Ferreira MU Kapilananda GMG Wirth DF Karunaweera ND, 2014 The Sri Lankan paradox: high genetic diversity in *Plasmodium vivax* populations despite decreasing levels of malaria transmission. Parasitology 141: 880–890. 24533989 10.1017/S0031182013002278 PMC7485621

[75] Noviyanti R 2015 Contrasting transmission dynamics of co-endemic *Plasmodium vivax* and *P. falciparum*: implications for malaria control and elimination. PLoS Negl Trop Dis 9: e0003739. 25951184 10.1371/journal.pntd.0003739 PMC4423885

[76] Neafsey DE Galinsky K Jiang RHY Young L Sykes SM Saif S Gujja S Goldberg JM Young S Zeng Q, 2012 The malaria parasite *Plasmodium vivax* exhibits greater genetic diversity than *Plasmodium falciparum*. Nat Genet 44: 1046–1050. 22863733 10.1038/ng.2373 PMC3432710

[77] Rosanas-Urgell A 2012 Reduced risk of *Plasmodium vivax* malaria in Papua New Guinean children with southeast Asian ovalocytosis in two cohorts and a case-control study. PLoS Med 9: e1001305. 22973182 10.1371/journal.pmed.1001305 PMC3433408

[78] Pearson RD 2016 Genomic analysis of local variation and recent evolution in *Plasmodium vivax*. Nat Genet 48: 959–964. 27348299 10.1038/ng.3599 PMC4966634

[79] Ménard D 2010 *Plasmodium vivax* clinical malaria is commonly observed in Duffy-negative Malagasy people. Proc Natl Acad Sci USA 107: 5967–5971. 20231434 10.1073/pnas.0912496107 PMC2851935

[80] Luo Z Sullivan SA Carlton JM, 2015 The biology of *Plasmodium vivax* explored through genomics. Ann N Y Acad Sci 1342: 53–61. 25693446 10.1111/nyas.12708 PMC4405435

[81] Ntumngia FB Thomson-Luque R Torres L de M Gunalan K Carvalho LH Adams JH, 2016 A novel erythrocyte binding protein of *Plasmodium vivax* suggests an alternate invasion pathway into Duffy-positive reticulocytes. MBio 7: e01261–16. 27555313 10.1128/mBio.01261-16 PMC4999553

[82] Price RN von Seidlein L Valecha N Nosten F Baird JK White NJ, 2014 Global extent of chloroquine-resistant *Plasmodium vivax*: a systematic review and meta-analysis. Lancet Infect Dis 14: 982–991. 25213732 10.1016/S1473-3099(14)70855-2 PMC4178238

[83] Douglas NM Anstey NM Angus BJ Nosten F Price RN, 2010 Artemisinin combination therapy for vivax malaria. Lancet Infect Dis 10: 405–416. 20510281 10.1016/S1473-3099(10)70079-7 PMC3350863

[84] William T Menon J Rajahram G Chan L Ma G Donaldson S Khoo S Fredrick C Jelip J Anstey NM, 2011 Severe *Plasmodium knowlesi* malaria in a tertiary care hospital, Sabah, Malaysia. Emerg Infect Dis 17: 1248. 21762579 10.3201/eid.1707.101017 PMC3381373

[85] Mueller I Zimmerman PA Reeder JC, 2007 *Plasmodium malariae* and *Plasmodium ovale*—the “bashful” malaria parasites. Trends Parasitol 23: 278–283. 17459775 10.1016/j.pt.2007.04.009 PMC3728836

[86] Home Page—ClinicalTrials.gov. Available at: https://clinicaltrials.gov/. Accessed August 25, 2017.

[87] Takeuchi R 2010 Directly-observed therapy (DOT) for the radical 14-day primaquine treatment of *Plasmodium vivax* malaria on the Thai-Myanmar border. Malar J 9: 308. 21040545 10.1186/1475-2875-9-308 PMC2988041

[88] John GK Douglas NM von Seidlein L Nosten F Baird JK White NJ Price RN, 2012 Primaquine radical cure of *Plasmodium vivax*: a critical review of the literature. Malar J 11: 280. 22900786 10.1186/1475-2875-11-280 PMC3489597

[89] Douglas NM Poespoprodjo JR Patriani D Malloy MJ Kenangalem E Sugiarto P Simpson JA Soenarto Y Anstey NM Price RN, 2017 Unsupervised primaquine for the treatment of *Plasmodium vivax* malaria relapses in southern Papua: a hospital-based cohort study. PLoS Med 14: e1002379. 28850568 10.1371/journal.pmed.1002379 PMC5574534

[90] Howes RE 2013 Spatial distribution of G6PD deficiency variants across malaria-endemic regions. Malar J 12: 418. 24228846 10.1186/1475-2875-12-418 PMC3835423

[91] Newby G 2015 Review of mass drug administration for malaria and its operational challenges. Am J Trop Med Hyg 93: 125–134. 26013371 10.4269/ajtmh.14-0254 PMC4497884

[92] Tseroni M 2015 Prevention of malaria resurgence in Greece through the association of mass drug administration (MDA) to immigrants from malaria-endemic regions and standard control measures. PLoS Negl Trop Dis 9: e0004215. 26583650 10.1371/journal.pntd.0004215 PMC4652894

[93] Krotoski WA, 1980 Frequency of relapse and primaquine resistance in southeast Asian vivax malaria. N Engl J Med 303: 587. 10.1056/NEJM198009043031022 6995839

[94] John CC, 2016 Primaquine plus artemisinin combination therapy for reduction of malaria transmission: promise and risk. BMC Med 14: 65. 27039396 10.1186/s12916-016-0611-9 PMC4818907

[95] Bennett JW Pybus BS Yadava A Tosh D Sousa JC McCarthy WF Deye G Melendez V Ockenhouse CF, 2013 Primaquine failure and cytochrome p-450 2D6 in *Plasmodium vivax* malaria. N Engl J Med 369: 1381–1382. 24088113 10.1056/NEJMc1301936

